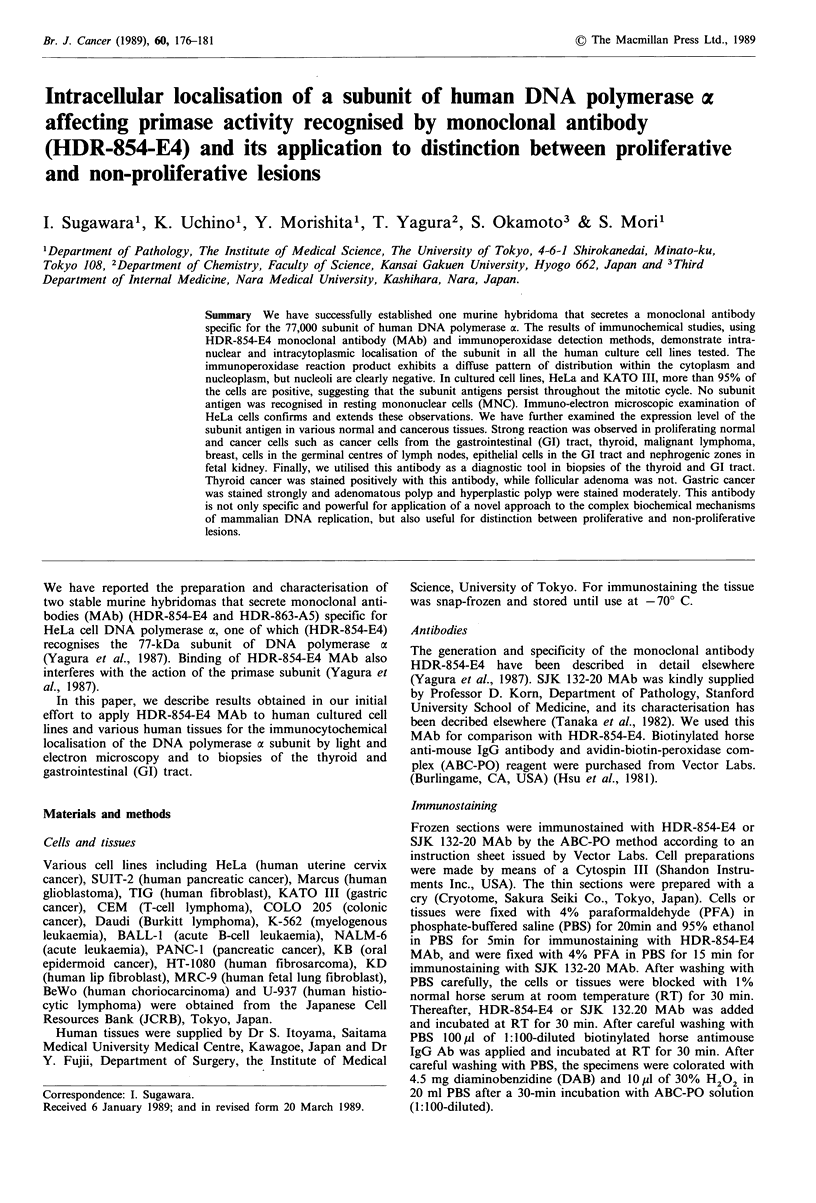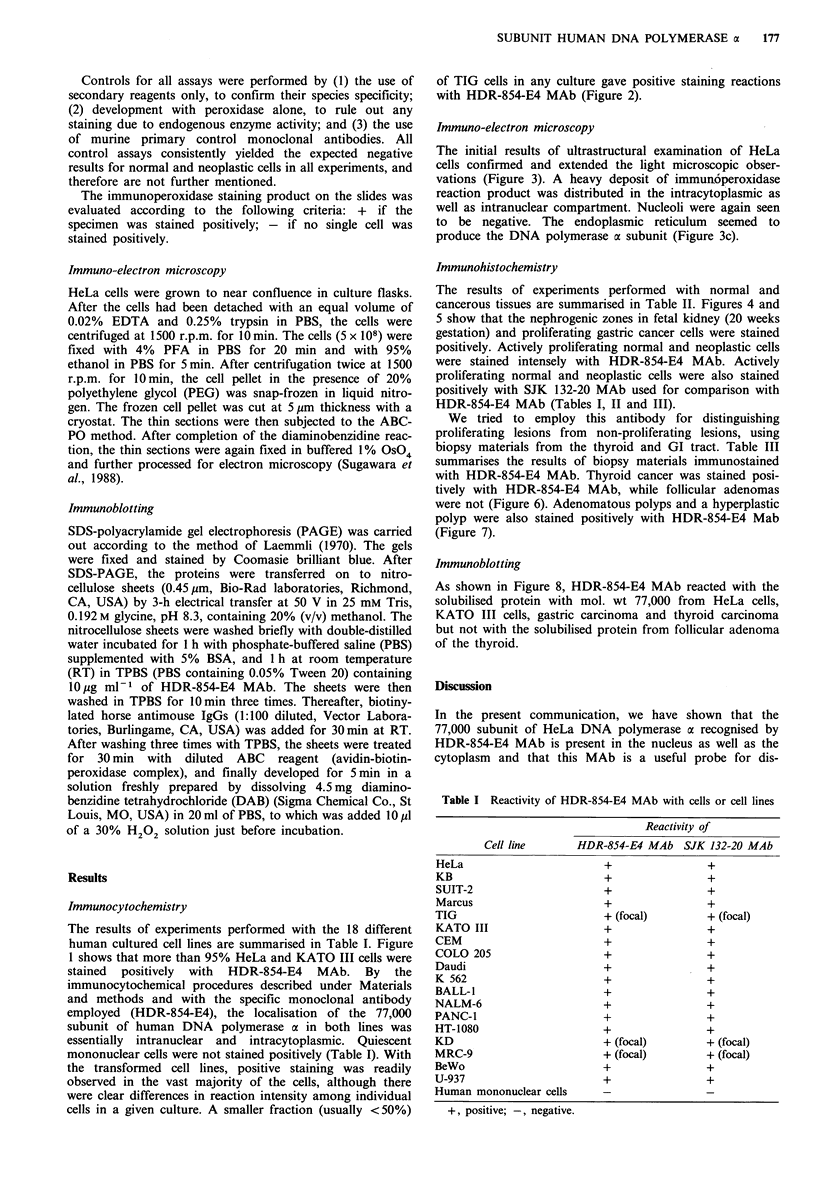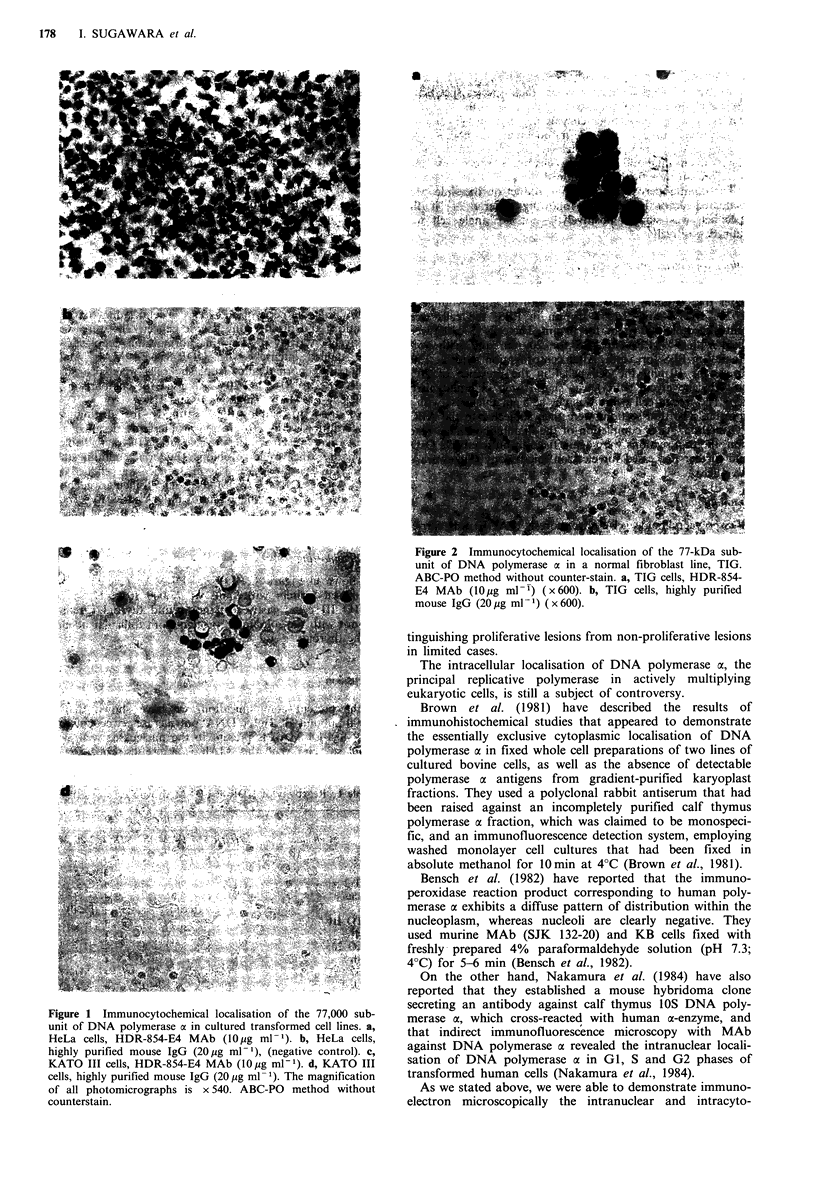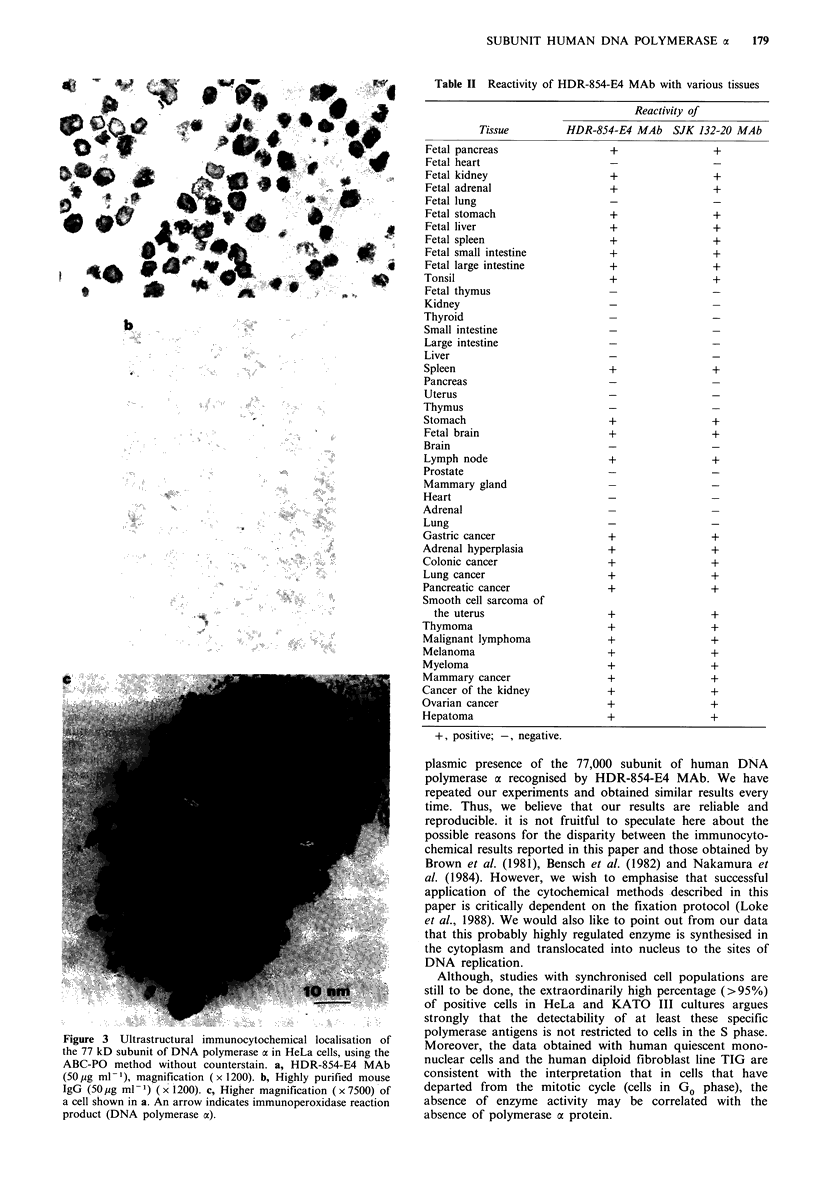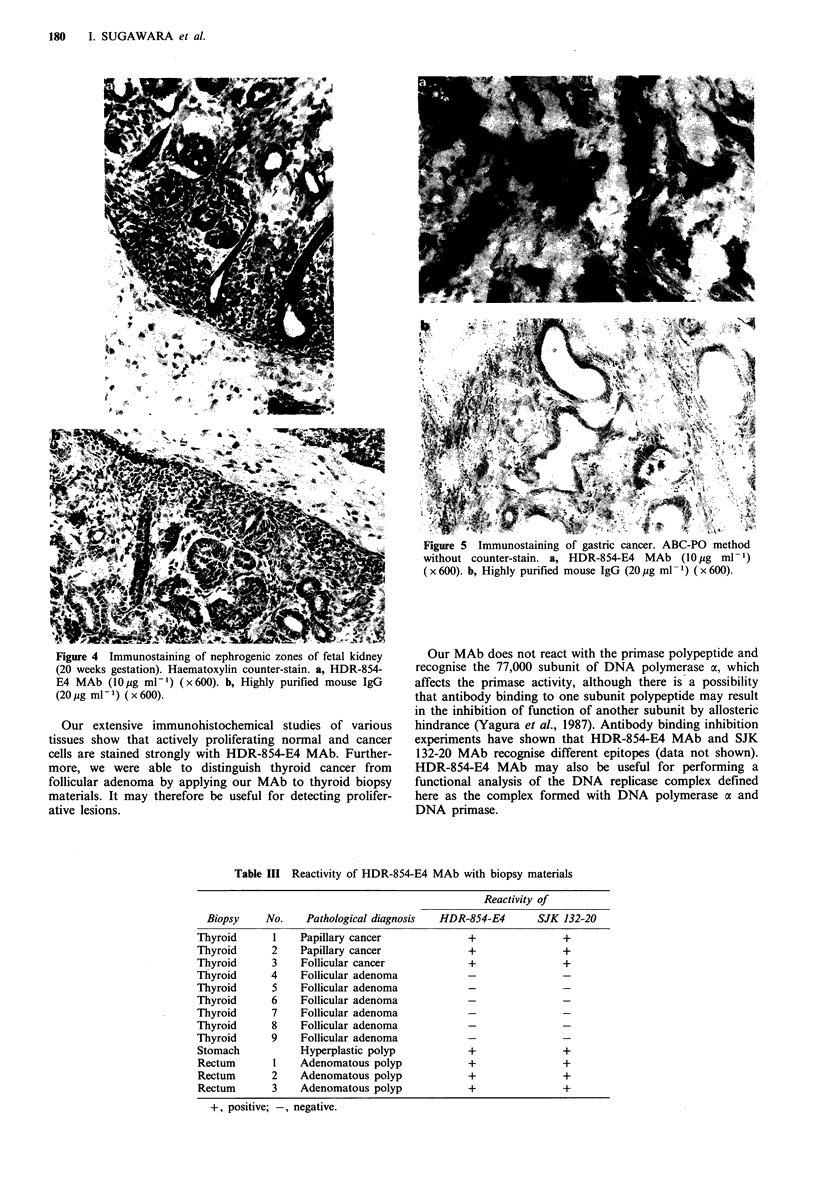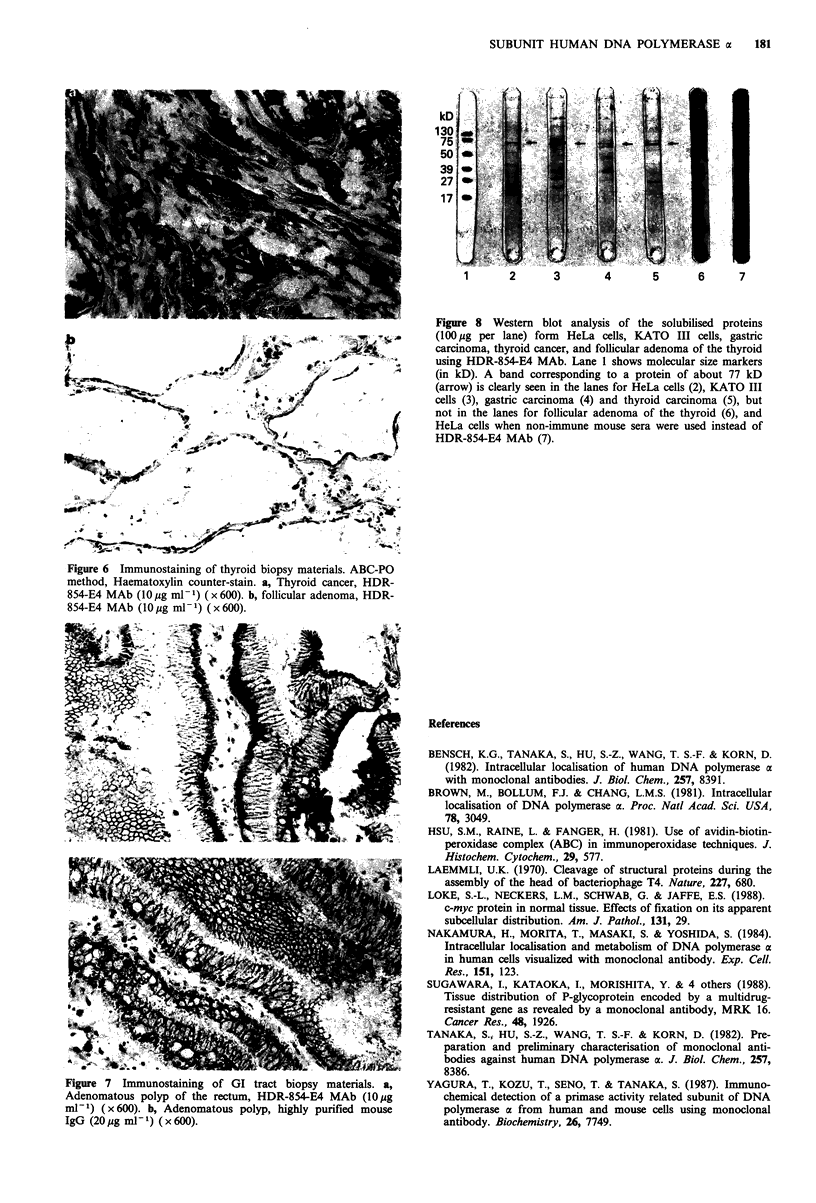# Intracellular localisation of a subunit of human DNA polymerase alpha affecting primase activity recognised by monoclonal antibody (HDR-854-E4) and its application to distinction between proliferative and non-proliferative lesions.

**DOI:** 10.1038/bjc.1989.246

**Published:** 1989-08

**Authors:** I. Sugawara, K. Uchino, Y. Morishita, T. Yagura, S. Okamoto, S. Mori

**Affiliations:** Department of Pathology, University of Tokyo, Japan.

## Abstract

**Images:**


*Br. J. Cancer* (1989), **60**, 176–181 © The Macmillan Press Ltd., 1989

### Intracellular localisation of a subunit of human DNA polymerase α affecting primase activity recognised by monoclonal antibody (HDR-854-E4) and its application to distinction between proliferative and non-proliferative lesions

I. Sugawara[1], K. Uchino[1], Y. Morishita[1], T. Yagura[2], S. Okamoto[3] & S. Mori[1]

[1]*Department of Pathology, The Institute of Medical Science, The University of Tokyo, 4-6-1 Shirokanedai, Minato-ku, Tokyo 108,* [2]*Department of Chemistry, Faculty of Science, Kansai Gakuen University, Hyogo 662, Japan and* [3]*Third Department of Internal Medicine, Nara Medical University, Kashihara, Nara, Japan.*

**Summary** We have successfully established one murine hybridoma that secretes a monoclonal antibody specific for the 77,000 subunit of human DNA polymerase α. The results of immunochemical studies, using HDR-854-E4 monoclonal antibody (MAb) and immunoperoxidase detection methods, demonstrate intranuclear and intracytoplasmic localisation of the subunit in all the human culture cell lines tested. The immunoperoxidase reaction product exhibits a diffuse pattern of distribution within the cytoplasm and nucleoplasm, but nucleoli are clearly negative. In cultured cell lines, HeLa and KATO III, more than 95% of the cells are positive, suggesting that the subunit antigens persist throughout the mitotic cycle. No subunit antigen was recognised in resting mononuclear cells (MNC). Immuno-electron microscopic examination of HeLa cells confirms and extends these observations. We have further examined the expression level of the subunit antigen in various normal and cancerous tissues. Strong reaction was observed in proliferating normal and cancer cells such as cancer cells from the gastrointestinal (GI) tract, thyroid, malignant lymphoma, breast, cells in the germinal centres of lymph nodes, epithelial cells in the GI tract and nephrogenic zones in fetal kidney. Finally, we utilised this antibody as a diagnostic tool in biopsies of the thyroid and GI tract. Thyroid cancer was stained positively with this antibody, while follicular adenoma was not. Gastric cancer was stained strongly and adenomatous polyp and hyperplastic polyp were stained moderately. This antibody is not only specific and powerful for application of a novel approach to the complex biochemical mechanisms of mammalian DNA replication, but also useful for distinction between proliferative and non-proliferative lesions.

We have reported the preparation and characterisation of two stable murine hybridomas that secrete monoclonal antibodies (MAb) (HDR-854-E4 and HDR-863-A5) specific for HeLa cell DNA polymerase α, one of which (HDR-854-E4) recognises the 77-kDa subunit of DNA polymerase α (Yagura *et al.*, 1987). Binding of HDR-854-E4 MAb also interferes with the action of the primase subunit (Yagura *et al.*, 1987).

In this paper, we describe results obtained in our initial effort to apply HDR-854-E4 MAb to human cultured cell lines and various human tissues for the immunocytochemical localisation of the DNA polymerase α subunit by light and electron microscopy and to biopsies of the thyroid and gastrointestinal (GI) tract.

### Materials and methods

### *Cells and tissues*

Various cell lines including HeLa (human uterine cervix cancer), SUIT-2 (human pancreatic cancer), Marcus (human glioblastoma), TIG (human fibroblast), KATO III (gastric cancer), CEM (T-cell lymphoma), COLO 205 (colonic cancer), Daudi (Burkitt lymphoma), K-562 (myelogenous leukaemia), BALL-1 (acute B-cell leukaemia), NALM-6 (acute leukaemia), PANC-1 (pancreatic cancer), KB (oral epidermoid cancer), HT-1080 (human fibrosarcoma), KD (human lip fibroblast), MRC-9 (human fetal lung fibroblast), BeWo (human choriocarcinoma) and U-937 (human histiocytic lymphoma) were obtained from the Japanese Cell Resources Bank (JCRB), Tokyo, Japan.

Human tissues were supplied by Dr S. Itoyama, Saitama Medical University Medical Centre, Kawagoe, Japan and Dr Y. Fujii, Department of Surgery, the Institute of Medical Science, University of Tokyo. For immunostaining the tissue was snap-frozen and stored until use at −70° C.

### *Antibodies*

The generation and specificity of the monoclonal antibody HDR-854-E4 have been described in detail elsewhere (Yagura *et al.*, 1987). SJK 132-20 MAb was kindly supplied by Professor D. Korn, Department of Pathology, Stanford University School of Medicine, and its characterisation has been decribed elsewhere (Tanaka *et al.*, 1982). We used this MAb for comparison with HDR-854-E4. Biotinylated horse anti-mouse IgG antibody and avidin-biotin-peroxidase complex (ABC-PO) reagent were purchased from Vector Labs. (Burlingame, CA, USA) (Hsu *et al.*, 1981).

### *Immunostaining*

Frozen sections were immunostained with HDR-854-E4 or SJK 132-20 MAb by the ABC-PO method according to an instruction sheet issued by Vector Labs. Cell preparations were made by means of a Cytospin III (Shandon Instruments Inc., USA). The thin sections were prepared with a cry (Cryotome, Sakura Seiki Co., Tokyo, Japan). Cells or tissues were fixed with 4% paraformaldehyde (PFA) in phosphate-buffered saline (PBS) for 20min and 95% ethanol in PBS for 5min for immunostaining with HDR-854-E4 MAb, and were fixed with 4% PFA in PBS for 15 min for immunostaining with SJK 132-20 MAb. After washing with PBS carefully, the cells or tissues were blocked with 1% normal horse serum at room temperature (RT) for 30 min. Thereafter, HDR-854-E4 or SJK 132.20 MAb was added and incubated at RT for 30 min. After careful washing with PBS 100 μl of 1:100-diluted biotinylated horse antimouse IgG Ab was applied and incubated at RT for 30 min. After careful washing with PBS, the specimens were colorated with 4.5 mg diaminobenzidine (DAB) and 10 μl of 30% $H_2O_2$ in 20 ml PBS after a 30-min incubation with ABC-PO solution (1:100-diluted).

Correspondence: I. Sugawara.
Received 6 January 1989; and in revised form 20 March 1989.

Controls for all assays were performed by (1) the use of secondary reagents only, to confirm their species specificity; (2) development with peroxidase alone, to rule out any staining due to endogenous enzyme activity; and (3) the use of murine primary control monoclonal antibodies. All control assays consistently yielded the expected negative results for normal and neoplastic cells in all experiments, and therefore are not further mentioned.

The immunoperoxidase staining product on the slides was evaluated according to the following criteria: + if the specimen was stained positively; − if no single cell was stained positively.

*Immuno-electron microscopy*

HeLa cells were grown to near confluence in culture flasks. After the cells had been detached with an equal volume of 0.02% EDTA and 0.25% trypsin in PBS, the cells were centrifuged at 1500 r.p.m. for 10 min. The cells ($5 \times 10^8$) were fixed with 4% PFA in PBS for 20 min and with 95% ethanol in PBS for 5 min. After centrifugation twice at 1500 r.p.m. for 10 min, the cell pellet in the presence of 20% polyethylene glycol (PEG) was snap-frozen in liquid nitrogen. The frozen cell pellet was cut at 5 μm thickness with a cryostat. The thin sections were then subjected to the ABC-PO method. After completion of the diaminobenzidine reaction, the thin sections were again fixed in buffered 1% $OsO_4$ and further processed for electron microscopy (Sugawara *et al.*, 1988).

*Immunoblotting*

SDS-polyacrylamide gel electrophoresis (PAGE) was carried out according to the method of Laemmli (1970). The gels were fixed and stained by Coomasie brilliant blue. After SDS-PAGE, the proteins were transferred on to nitrocellulose sheets (0.45 μm, Bio-Rad laboratories, Richmond, CA, USA) by 3-h electrical transfer at 50 V in 25 mM Tris, 0.192 M glycine, pH 8.3, containing 20% (v/v) methanol. The nitrocellulose sheets were washed briefly with double-distilled water incubated for 1 h with phosphate-buffered saline (PBS) supplemented with 5% BSA, and 1 h at room temperature (RT) in TPBS (PBS containing 0.05% Tween 20) containing 10 μg $ml^{-1}$ of HDR-854-E4 MAb. The sheets were then washed in TPBS for 10 min three times. Thereafter, biotinylated horse antimouse IgGs (1:100 diluted, Vector Laboratories, Burlingame, CA, USA) was added for 30 min at RT. After washing three times with TPBS, the sheets were treated for 30 min with diluted ABC reagent (avidin-biotin-peroxidase complex), and finally developed for 5 min in a solution freshly prepared by dissolving 4.5 mg diaminobenzidine tetrahydrochloride (DAB) (Sigma Chemical Co., St Louis, MO, USA) in 20 ml of PBS, to which was added 10 μl of a 30% $H_2O_2$ solution just before incubation.

### Results

*Immunocytochemistry*

The results of experiments performed with the 18 different human cultured cell lines are summarised in Table I. Figure 1 shows that more than 95% HeLa and KATO III cells were stained positively with HDR-854-E4 MAb. By the immunocytochemical procedures described under Materials and methods and with the specific monoclonal antibody employed (HDR-854-E4), the localisation of the 77,000 subunit of human DNA polymerase α in both lines was essentially intranuclear and intracytoplasmic. Quiescent mononuclear cells were not stained positively (Table I). With the transformed cell lines, positive staining was readily observed in the vast majority of the cells, although there were clear differences in reaction intensity among individual cells in a given culture. A smaller fraction (usually <50%) of TIG cells in any culture gave positive staining reactions with HDR-854-E4 MAb (Figure 2).

*Immuno-electron microscopy*

The initial results of ultrastructural examination of HeLa cells confirmed and extended the light microscopic observations (Figure 3). A heavy deposit of immunoperoxidase reaction product was distributed in the intracytoplasmic as well as intranuclear compartment. Nucleoli were again seen to be negative. The endoplasmic reticulum seemed to produce the DNA polymerase α subunit (Figure 3c).

*Immunohistochemistry*

The results of experiments performed with normal and cancerous tissues are summarised in Table II. Figures 4 and 5 show that the nephrogenic zones in fetal kidney (20 weeks gestation) and proliferating gastric cancer cells were stained positively. Actively proliferating normal and neoplastic cells were stained intensely with HDR-854-E4 MAb. Actively proliferating normal and neoplastic cells were also stained positively with SJK 132-20 MAb used for comparison with HDR-854-E4 MAb (Tables I, II and III).

We tried to employ this antibody for distinguishing proliferating lesions from non-proliferating lesions, using biopsy materials from the thyroid and GI tract. Table III summarises the results of biopsy materials immunostained with HDR-854-E4 MAb. Thyroid cancer was stained positively with HDR-854-E4 MAb, while follicular adenomas were not (Figure 6). Adenomatous polyps and a hyperplastic polyp were also stained positively with HDR-854-E4 Mab (Figure 7).

*Immunoblotting*

As shown in Figure 8, HDR-854-E4 MAb reacted with the solubilised protein with mol. wt 77,000 from HeLa cells, KATO III cells, gastric carcinoma and thyroid carcinoma but not with the solubilised protein from follicular adenoma of the thyroid.

### Discussion

In the present communication, we have shown that the 77,000 subunit of HeLa DNA polymerase α recognised by HDR-854-E4 MAb is present in the nucleus as well as the cytoplasm and that this MAb is a useful probe for dis-

**Table I** Reactivity of HDR-854-E4 MAb with cells or cell lines

| | Reactivity of | |
|---|---|---|
| *Cell line* | *HDR-854-E4 MAb* | *SJK 132-20 MAb* |
| HeLa | + | + |
| KB | + | + |
| SUIT-2 | + | + |
| Marcus | + | + |
| TIG | + (focal) | + (focal) |
| KATO III | + | + |
| CEM | + | + |
| COLO 205 | + | + |
| Daudi | + | + |
| K 562 | + | + |
| BALL-1 | + | + |
| NALM-6 | + | + |
| PANC-1 | + | + |
| HT-1080 | + | + |
| KD | + (focal) | + (focal) |
| MRC-9 | + (focal) | + (focal) |
| BeWo | + | + |
| U-937 | + | + |
| Human mononuclear cells | − | − |

+, positive; −, negative.

**Figure 1** Immunocytochemical localisation of the 77,000 subunit of DNA polymerase $\alpha$ in cultured transformed cell lines. **a,** HeLa cells, HDR-854-E4 MAb (10 $\mu$g ml$^{-1}$). **b,** HeLa cells, highly purified mouse IgG (20 $\mu$g ml$^{-1}$), (negative control). **c,** KATO III cells, HDR-854-E4 MAb (10 $\mu$g ml$^{-1}$). **d,** KATO III cells, highly purified mouse IgG (20 $\mu$g ml$^{-1}$). The magnification of all photomicrographs is $\times 540$. ABC-PO method without counterstain.

**Figure 2** Immunocytochemical localisation of the 77-kDa subunit of DNA polymerase $\alpha$ in a normal fibroblast line, TIG. ABC-PO method without counter-stain. **a,** TIG cells, HDR-854-E4 MAb (10 $\mu$g ml$^{-1}$) ($\times 600$). **b,** TIG cells, highly purified mouse IgG (20 $\mu$g ml$^{-1}$) ($\times 600$).

tinguishing proliferative lesions from non-proliferative lesions in limited cases.

The intracellular localisation of DNA polymerase $\alpha$, the principal replicative polymerase in actively multiplying eukaryotic cells, is still a subject of controversy.

Brown *et al.* (1981) have described the results of immunohistochemical studies that appeared to demonstrate the essentially exclusive cytoplasmic localisation of DNA polymerase $\alpha$ in fixed whole cell preparations of two lines of cultured bovine cells, as well as the absence of detectable polymerase $\alpha$ antigens from gradient-purified karyoplast fractions. They used a polyclonal rabbit antiserum that had been raised against an incompletely purified calf thymus polymerase $\alpha$ fraction, which was claimed to be monospecific, and an immunofluorescence detection system, employing washed monolayer cell cultures that had been fixed in absolute methanol for 10 min at 4°C (Brown *et al.*, 1981).

Bensch *et al.* (1982) have reported that the immunoperoxidase reaction product corresponding to human polymerase $\alpha$ exhibits a diffuse pattern of distribution within the nucleoplasm, whereas nucleoli are clearly negative. They used murine MAb (SJK 132-20) and KB cells fixed with freshly prepared 4% paraformaldehyde solution (pH 7.3; 4°C) for 5–6 min (Bensch *et al.*, 1982).

On the other hand, Nakamura *et al.* (1984) have also reported that they established a mouse hybridoma clone secreting an antibody against calf thymus 10S DNA polymerase $\alpha$, which cross-reacted with human $\alpha$-enzyme, and that indirect immunofluorescence microscopy with MAb against DNA polymerase $\alpha$ revealed the intranuclear localisation of DNA polymerase $\alpha$ in G1, S and G2 phases of transformed human cells (Nakamura *et al.*, 1984).

As we stated above, we were able to demonstrate immunoelectron microscopically the intranuclear and intracyto-

**Figure 3** Ultrastructural immunocytochemical localisation of the 77 kD subunit of DNA polymerase α in HeLa cells, using the ABC-PO method without counterstain. **a**, HDR-854-E4 MAb (50 $\mu$g ml$^{-1}$), magnification (×1200). **b**, Highly purified mouse IgG (50 $\mu$g ml$^{-1}$) (×1200). **c**, Higher magnification (×7500) of a cell shown in **a**. An arrow indicates immunoperoxidase reaction product (DNA polymerase α).

**Table II** Reactivity of HDR-854-E4 MAb with various tissues

| *Tissue* | *Reactivity of* HDR-854-E4 MAb | *Reactivity of* SJK 132-20 MAb |
|---|---|---|
| Fetal pancreas | + | + |
| Fetal heart | − | − |
| Fetal kidney | + | + |
| Fetal adrenal | + | + |
| Fetal lung | − | − |
| Fetal stomach | + | + |
| Fetal liver | + | + |
| Fetal spleen | + | + |
| Fetal small intestine | + | + |
| Fetal large intestine | + | + |
| Tonsil | + | + |
| Fetal thymus | − | − |
| Kidney | − | − |
| Thyroid | − | − |
| Small intestine | − | − |
| Large intestine | − | − |
| Liver | − | − |
| Spleen | + | + |
| Pancreas | − | − |
| Uterus | − | − |
| Thymus | − | − |
| Stomach | + | + |
| Fetal brain | + | + |
| Brain | − | − |
| Lymph node | + | + |
| Prostate | − | − |
| Mammary gland | − | − |
| Heart | − | − |
| Adrenal | − | − |
| Lung | − | − |
| Gastric cancer | + | + |
| Adrenal hyperplasia | + | + |
| Colonic cancer | + | + |
| Lung cancer | + | + |
| Pancreatic cancer | + | + |
| Smooth cell sarcoma of the uterus | + | + |
| Thymoma | + | + |
| Malignant lymphoma | + | + |
| Melanoma | + | + |
| Myeloma | + | + |
| Mammary cancer | + | + |
| Cancer of the kidney | + | + |
| Ovarian cancer | + | + |
| Hepatoma | + | + |

+, positive; −, negative.

plasmic presence of the 77,000 subunit of human DNA polymerase α recognised by HDR-854-E4 MAb. We have repeated our experiments and obtained similar results every time. Thus, we believe that our results are reliable and reproducible. it is not fruitful to speculate here about the possible reasons for the disparity between the immunocytochemical results reported in this paper and those obtained by Brown *et al.* (1981), Bensch *et al.* (1982) and Nakamura *et al.* (1984). However, we wish to emphasise that successful application of the cytochemical methods described in this paper is critically dependent on the fixation protocol (Loke *et al.*, 1988). We would also like to point out from our data that this probably highly regulated enzyme is synthesised in the cytoplasm and translocated into nucleus to the sites of DNA replication.

Although, studies with synchronised cell populations are still to be done, the extraordinarily high percentage (>95%) of positive cells in HeLa and KATO III cultures argues strongly that the detectability of at least these specific polymerase antigens is not restricted to cells in the S phase. Moreover, the data obtained with human quiescent mononuclear cells and the human diploid fibroblast line TIG are consistent with the interpretation that in cells that have departed from the mitotic cycle (cells in $G_0$ phase), the absence of enzyme activity may be correlated with the absence of polymerase α protein.

**Figure 4** Immunostaining of nephrogenic zones of fetal kidney (20 weeks gestation). Haematoxylin counter-stain. **a,** HDR-854-E4 MAb (10 $\mu$g ml$^{-1}$) ($\times$600). **b,** Highly purified mouse IgG (20 $\mu$g ml$^{-1}$) ($\times$600).

**Figure 5** Immunostaining of gastric cancer. ABC-PO method without counter-stain. **a,** HDR-854-E4 MAb (10 $\mu$g ml$^{-1}$) ($\times$600). **b,** Highly purified mouse IgG (20 $\mu$g ml$^{-1}$) ($\times$600).

Our extensive immunohistochemical studies of various tissues show that actively proliferating normal and cancer cells are stained strongly with HDR-854-E4 MAb. Furthermore, we were able to distinguish thyroid cancer from follicular adenoma by applying our MAb to thyroid biopsy materials. It may therefore be useful for detecting proliferative lesions.

Our MAb does not react with the primase polypeptide and recognise the 77,000 subunit of DNA polymerase $\alpha$, which affects the primase activity, although there is a possibility that antibody binding to one subunit polypeptide may result in the inhibition of function of another subunit by allosteric hindrance (Yagura *et al.*, 1987). Antibody binding inhibition experiments have shown that HDR-854-E4 MAb and SJK 132-20 MAb recognise different epitopes (data not shown). HDR-854-E4 MAb may also be useful for performing a functional analysis of the DNA replicase complex defined here as the complex formed with DNA polymerase $\alpha$ and DNA primase.

**Table III** Reactivity of HDR-854-E4 MAb with biopsy materials

| | | | *Reactivity of* | |
|---|---|---|---|---|
| *Biopsy* | *No.* | *Pathological diagnosis* | *HDR-854-E4* | *SJK 132-20* |
| Thyroid | 1 | Papillary cancer | + | + |
| Thyroid | 2 | Papillary cancer | + | + |
| Thyroid | 3 | Follicular cancer | + | + |
| Thyroid | 4 | Follicular adenoma | − | − |
| Thyroid | 5 | Follicular adenoma | − | − |
| Thyroid | 6 | Follicular adenoma | − | − |
| Thyroid | 7 | Follicular adenoma | − | − |
| Thyroid | 8 | Follicular adenoma | − | − |
| Thyroid | 9 | Follicular adenoma | − | − |
| Stomach | | Hyperplastic polyp | + | + |
| Rectum | 1 | Adenomatous polyp | + | + |
| Rectum | 2 | Adenomatous polyp | + | + |
| Rectum | 3 | Adenomatous polyp | + | + |

+, positive; −, negative.

**Figure 6** Immunostaining of thyroid biopsy materials. ABC-PO method, Haematoxylin counter-stain. **a,** Thyroid cancer, HDR-854-E4 MAb (10 μg ml$^{-1}$) (×600). **b,** follicular adenoma, HDR-854-E4 MAb (10 μg ml$^{-1}$) (×600).

**Figure 7** Immunostaining of GI tract biopsy materials. **a,** Adenomatous polyp of the rectum, HDR-854-E4 MAb (10 μg ml$^{-1}$) (×600). **b,** Adenomatous polyp, highly purified mouse IgG (20 μg ml$^{-1}$) (×600).

**Figure 8** Western blot analysis of the solubilised proteins (100 μg per lane) form HeLa cells, KATO III cells, gastric carcinoma, thyroid cancer, and follicular adenoma of the thyroid using HDR-854-E4 MAb. Lane 1 shows molecular size markers (in kD). A band corresponding to a protein of about 77 kD (arrow) is clearly seen in the lanes for HeLa cells (2), KATO III cells (3), gastric carcinoma (4) and thyroid carcinoma (5), but not in the lanes for follicular adenoma of the thyroid (6), and HeLa cells when non-immune mouse sera were used instead of HDR-854-E4 MAb (7).

### References

BENSCH, K.G., TANAKA, S., HU, S.-Z., WANG, T. S.-F. & KORN, D. (1982). Intracellular localisation of human DNA polymerase α with monoclonal antibodies. *J. Biol. Chem.*, **257,** 8391.

BROWN, M., BOLLUM, F.J. & CHANG, L.M.S. (1981). Intracellular localisation of DNA polymerase α. *Proc. Natl Acad. Sci. USA*, **78,** 3049.

HSU, S.M., RAINE, L. & FANGER, H. (1981). Use of avidin-biotin-peroxidase complex (ABC) in immunoperoxidase techniques. *J. Histochem. Cytochem.*, **29,** 577.

LAEMMLI, U.K. (1970). Cleavage of structural proteins during the assembly of the head of bacteriophage T4. *Nature*, **227,** 680.

LOKE, S.-L., NECKERS, L.M., SCHWAB, G. & JAFFE, E.S. (1988). c-*myc* protein in normal tissue. Effects of fixation on its apparent subcellular distribution. *Am. J. Pathol.*, **131,** 29.

NAKAMURA, H., MORITA, T., MASAKI, S. & YOSHIDA, S. (1984). Intracellular localisation and metabolism of DNA polymerase α in human cells visualized with monoclonal antibody. *Exp. Cell. Res.*, **151,** 123.

SUGAWARA, I., KATAOKA, I., MORISHITA, Y. & 4 others (1988). Tissue distribution of P-glycoprotein encoded by a multidrug-resistant gene as revealed by a monoclonal antibody, MRK 16. *Cancer Res.*, **48,** 1926.

TANAKA, S., HU, S.-Z., WANG, T. S.-F. & KORN, D. (1982). Preparation and preliminary characterisation of monoclonal antibodies against human DNA polymerase α. *J. Biol. Chem.*, **257,** 8386.

YAGURA, T., KOZU, T., SENO, T. & TANAKA, S. (1987). Immunochemical detection of a primase activity related subunit of DNA polymerase α from human and mouse cells using monoclonal antibody. *Biochemistry*, **26,** 7749.